# Evaluation of Paeonol Skin-Target Delivery from Its Microsponge Formulation: *In Vitro* Skin Permeation and *In Vivo* Microdialysis

**DOI:** 10.1371/journal.pone.0079881

**Published:** 2013-11-20

**Authors:** Sha-Sha Li, Guo-Feng Li, Li Liu, Xiao Jiang, Bin Zhang, Zhi-Gang Liu, Xue-Ling Li, Li-Dong Weng, Ting Zuo, Qiang Liu

**Affiliations:** 1 Nanfang Hospital, Southern Medical University, Guangzhou, PR China; 2 School of Traditional Chinese Medicine, Southern Medical University, Guangzhou, PR China; University Hospital Hamburg-Eppendorf, Germany

## Abstract

The aim of the present study was to design a novel topical skin-target drug-delivery system, the paeonol microsponge, and to investigate its drug-release patterns in dosage form, both *in vitro* and *in vivo*. Paeonol microsponges were prepared using the quasi-emulsion solvent-diffusion method. *In vitro* release studies were carried out using Franz diffusion cells, while *in vivo* studies were investigated by microdialysis after the paeonol microsponges were incorporated into a cream base. *In vitro* release studies showed that the drug delivered via microsponges increased the paeonol permeation rate. *Ex vivo* drug-deposition studies showed that the microsponge formulation improved drug residence in skin. In addition, *in vivo* microdialysis showed that the values for the area under the concentration versus time curve (AUC) for the paeonol microsponge cream was much higher than that of paeonol cream without microsponges. Maximum time (T_max_) was 220 min for paeonol microsponge cream and 480 min for paeonol cream, while the half-life (t_1/2_) of paeonol microsponge cream (935.1 min) was almost twice that of paeonol cream (548.6 min) in the skin (n = 3). Meanwhile, in the plasma, the AUC value for paeonol microsponge cream was half that of the paeonol cream. Based on these results, paeonol-loaded microsponge formulations could be a better alternative for treating skin disease, as the formulation increases drug bioavailability by lengthening the time of drug residence in the skin and should reduce side-effects because of the lower levels of paeonol moving into the circulation.

## Introduction

Controlled release of drugs into the epidermis often results in large amounts of the drug remaining at the delivery site, whereas much smaller amounts of the drug enter the body system. Although some transdermal delivery systems can be efficient in supplying drugs that have a systemic effect, they are not practical for controlling the delivery of drugs when the final target is the skin itself [1]. Thus, it would be useful to develop a delivery system that could maximize the period of drug deposit, either in the epidermis or the dermis, while minimizing its transdermal penetration into the body [2]. Microsponge has been increasingly investigated to reach the aim in recent years. Microsponges are a polymeric delivery system consisting of porous microspheres, and they may enhance the rate of dissolution of poorly water-soluble drugs by entrapping such drugs in microsponge pores. These pores are very small, and the drug can be reduced to microscopic particles in these pores, which significantly increase the surface area of the drug and increase the rate of solubility. Hence, this system may improve the efficacy and bioavailability of some poorly soluble drugs [1]. Microsponges are also designed to deliver active ingredients efficiently at minimum dose and to enhance stability, reduce side-effects, and modify drug release [2], [3]. Thus, the microsponge delivery system may be a promising drug-delivery system in treating skin disease.

Paeonol is one of the main active components from the root bark of *Paeonia suffruticosa* (the tree peony), which has been widely used in Asia and Europe. Paeonol has potential for the treatment of neurodegenerative diseases in humans by alleviating morphological damage [4], increasing neuron viability [5], and reducing cerebral infarction [5], [6]. Paeonol also possesses anti-atherogenic [7] and anti-arrhythmic [8] activities, and is widely used in cardiovascular diseases [9], [10]. Paeonol also has anti-tumor [11] and anti-inflammatory activity [12], as well as the ability to inhibit melanin [13]. Recently, it has been reported that paeonol has anti-anaphylactic activity through regulating histamine and tumor necrosis factor-α [14], and these effects could be exploited in treating eczema. However, paeonol is hydrophobic and has a low aqueous solubility, with an oil/water partition coefficient of 2.21 [13], [15]. Consequently, it is likely that it will be unable to penetrate the stratum corneum, and using enhancers may cause the drug to reach the blood too easily, but it is not desirable for the side effect. We know that, for any drug, including paeonol, to be effective in treating eczema, there must be a sufficient concentration of the drug in the epidermis. It is likely that paeonol would not be able to have an effect in treating skin disease, not because of the inefficacy of the drug itself, but because the drug cannot reach its site of action. Furthermore, increasing the amount of paeonol in the plasma might induce production of drug-metabolizing enzymes by the liver, which would affect the metabolism of other drugs. Therefore, formulation of a drug-delivery system to increase the rate of solubility of paeonol and its deposition in the epidermis, as well as to reduce its systemic action in patients with eczema, is of interest.

Previous authors have developed paeonol-loaded liposomes to increase paeonol bioavailability in skin tissues [16]. However, liposomes are very expensive and difficult to formulate in the final product and their manufacturing processes are very complex, thus it is difficult to carry out large-scale production with liposomes. Rui-guang et al did not report whether the prepared liposomes were stable over a long period at room temperature. Complexing paeonol with β-cyclodextrin was shown to increase its solubility [13], [15], but this formulation could not control the release rate of the active agent by itself. Recent studies have shown that microsponge formulations were able to deliver drugs to the colon and then release the drug while retained in the colonic lumen, indicating that this formulation could be a new approach for colon-specific drug delivery [3], [17]–[19]. In studies of a microsponge intra-dermal drug-delivery system, researchers used mupirocin, hydroxyzine hydrochloride, and benzoyl peroxide as the model drugs to evaluate the characteristics of this formulation, and showed that the main mechanism of drug release was diffusion and that the microsponge formulation could enhance the rate of drug release [2], [20]–[22]. Generally, microsponges were prepared mainly by a quasi-emulsion solvent-diffusion method and free-radical suspension polymerization [1]. However, free-radical suspension polymerization requires irradiation, high temperature, or catalysis to activate the monomers, and thus the manufacturing processes are very complex. In our project, the authors used an emulsion solvent-diffusion method to prepare the paeonol microsponges. Generally, the microsponges were stable over a wide range of pH values (1 to 11) and temperature (up to 130°C), and were compatible with most vehicles and ingredients. Because of the small pore diameter of the microsponges, bacteria cannot penetrate inside and reach their contents, thus, unlike liposomes and β-cyclodextrin, microsponges do not require any preservative compounds [23].

It has been shown that some microsponge formulations can significantly maximize the time that the active ingredient remains in the skin, while minimizing its penetration through the dermis into the body. In addition, the controlled release of the drug from the microsponge formulation into the epidermis means that the drug remains primarily localized, with only a restricted amount entering the systemic circulation, and thus can be used as a means of controlling side-effects [22]. Therefore, development of a microsponge delivery system for paeonol could improve drug efficacy in the local region and reduce side-effects [21].

In this study, we prepared paeonol microsponges using an emulsion solvent-diffusion method. Scanning electron microscopy (SEM), production yield, loading efficiency, and particle size distribution were used to evaluate the characteristic of the paeonol microsponges thus formed. The microsponges were then incorporated into a cream base and we carried out *in vitro* and *in vivo* drug-release studies.

## Materials and Methods

### Materials

Paeonol was purchased from Sigma Co. Ltd. (Tianjin, China). Ethyl cellulose-M70, polyvinyl alcohol (PVA) 1788, white beeswax, and stearic acid were obtained from Aladdin Chemistry Co. Ltd. (Shanghai, China). Triethanolamine and liquid paraffin were obtained from Tianjin Fuyu Chemical Reagents (Tianjin, China). All other chemicals and solvents were analytical grade.

### Animals

Female Wistar rats weighing about 200 g and female nude mice aged 6 to 7 weeks were purchased from the Center of Experimental Animals, Southern Medical University (Guangzhou, China). All animal experiments were performed according to the guidelines of the Experimental Animal Ethics Committee of Southern Medical University. And the protocol was approved by the Experimental Animal Ethics Committee of Southern Medical University (Permit Number for rats: 4402101707, Permit Number for nude mice: 4402102090). All surgery was performed under 10% chloral hydrate anesthesia, and all efforts were made to minimize suffering.

### Preparation of paeonol microsponges

Preparation was optimized based on production yield, loading efficiency and sorting coefficient (data not shown). The preparation procedure was as follows. The paeonol microsponges were prepared using a quasi-emulsion solvent-diffusion method with external and internal phases. To prepare the internal phase, paeonol 7 g and ethylcellulose 1 g were dissolved in dichloromethane 20 ml. In this procedure, dichloromethane was an effective solvent for dissolving both the drug and the polymer. The external phase, which contained the emulsifying agent PVA 3 g dissolved in 100 ml of distilled water, was placed in the vessel, and stirred with a propeller-type agitator at 1000 rpm, and then the internal phase was gradually added into the stirring external phase. The mixture was then stirred at 1000 rpm for 4 h at room temperature to remove the dichloromethane from the reaction flask. After that, the formed microsponges were filtered through filter paper with a pore size of 0.45 µm (Millipore, Maidstone, Kent, UK), washed with distilled water, and dried at room temperature.

### Analytical system

The quantitative determination of paeonol in microsponges was carried out using reverse-phase high-performance liquid chromatography (HPLC) using a chromatograph equipped with a quaternary pump (both model series 1100; Agilent Technologies Inc., Wilmington, DE, USA), on-line vacuum degasser, autosampler, column temperature controller, diode array, and multiple-wavelength detectors, along with an analytical workstation. The chromatographic separations were performed using an HPLC column 250×4.6 mm with a particle size of 5 µm (Syncronis C18; Thermo Fisher Scientific Inc., Rockford, IL, USA). A mixture of methanol and distilled water (65∶45) was used as the mobile phase. The filtered mobile phase was pumped at a flow rate of 1 ml/min, and the wavelength was set at 276 nm. All the determinations were performed at 25°C. The retention time for paeonol was found to be about 8.3 min, and the total run time was 10 min. A good linear relationship was found between the peak areas for various concentrations, from 0.004 mg/ml to 0.02 mg/ml (R^2^ = 0.9996). External standardization by peak area was used for quantitative determination of the paeonol microsponges. The developed method had good precision (0.114%) and accuracy (2.76%). Each determination was calculated in triplicate, and the mean of the values were reported.

### Characterization and evaluation of microsponge formulations

#### Scanning electron microscopy

The morphology and appearance of the microsponges were studied using SEM with a system (H-3000N, Japan) operating at 10 kV. The samples were dusted onto double-sided tape on a metal stub and coated with gold/palladium alloy under vacuum. The obtained photograph was recorded at ×400 magnification.

#### Determination of production yield

The production yield of the microparticles was calculated according to the following equation:
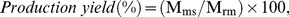
(1)where M_ms_ is the final weight of the microsponges obtained, and M_rm_ is the initial weight of the raw materials (polymer and drug). All the experiments were performed in triplicate and the mean of the values were reported.

#### Determination of drug content and loading efficiency

The quantitative determination of paeonol in microparticles was carried out using HPLC as described above. The drug-loaded microsponges were weighed accurately and treated with ultrasonic waves for 30 min with methanol as the extraction reagent. Before injection into the HPLC, the samples were filtered through a nylon membrane filter (0.45 µm). The actual drug content and loading efficiency were calculated according to the following equation [3]:
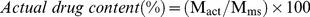
(2)

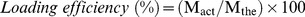
(3)where M_act_ is the actual quantity of paeonol in the weighed quantity of microparticles, M_ms_ is the weighed quantity of the microsponges, and M_the_ is the theoretical amount of paeonol in the microsponges.

#### Particle size distribution analysis

The particle size distribution of the microsponges was determined by a laser light-scattering technique (Mastersizer 2000; Malvern Instuments Ltd., Malvern, Worcestershire, UK). Before measurement, samples were dispersed in distilled water. The particle size range was set to 0.02 to 2000 µm, and the particle refractive index was set to 1.520. Sorting coefficient (σ) was calculated to evaluate particle uniformity, and particle size distribution by volume of the paeonol microsponge was calculated internally.

### Preparation of paeonol microsponge creams

Creams were prepared using a standard reverse-emulsification method, using an oil phase and an aqueous phase in order to carry out convenient administration of the microsponges to the skin. The aqueous phase, contained 3.7 g triethanolamine, and the oil phase consisted of 7 g stearic acid, 3.5 g liquid paraffin, and 1 g white beeswax. Both phases were heated to 65°C in a water bath. The aqueous phase was then added dropwise into the oil phase while the mixture was stirred using a magnetic stirrer, while being allowed to cool to room temperature, thus forming a cream. Once the cream was complete, we added either paeonol alone or the paeonol-containing microsponges to the cream, and thoroughly mixed the compound to ensure a homogenous preparation. As a comparison, a saturated solution of paeonol, using normal saline as solvent, was also prepared. In our study, the content of paeonol in the microsponge cream or paeonol cream was set at 50 mg/g to keep the cream stable (data not shown), while it reached 505.52 µg/ml in the saturated solution.

### *In vitro* drug-release studies

The *in vitro* release studies were carried out using a recirculating water bath and three Franz diffusion cells, with a receptor compartment volume of 15 mL and an effective area of 3.14 cm^2^. The permeation membrane was mouse skin, obtained from female nude mice aged 6–7 weeks. After the mouse was euthanized, the whole skin was excised, the subcutaneous fat was carefully removed with forceps, and then the skin was washed with normal saline and examined for integrity. The skin was clamped between the donor and the receptor chambers. The receptor chamber was filled with normal saline and set at 37°C, and then the solution in the receptor chambers was stirred continuously at 300 rpm. The respective formulation (paeonol cream 1.0 g, paeonol microsponge cream 1.0 g, or saturated aqueous solution 1 ml) was gently placed in the donor chamber. At 15 min, 30 min, 45 min, 1 h, 1.5 h, 2 h, 3 h, 4 h, 5 h, 6 h, 7 h, 8 h, 9 h, 10 h, 11 h, and 12 h, samples of 1 ml were withdrawn from the receiver compartment and replaced immediately with an equal volume of normal saline kept at 37°C. The collected samples were then analyzed by HPLC. The cumulative curve was plotted of the total amount of paeonol that permeated at each time interval *vs.* time. The release kinetics of the paeonol cream and paeonol microsponge cream were calculated, and their release patterns were analyzed using different mathematical modes.

### *Ex vivo* drug-deposition studies

For determination of the amount of drug deposited in the skin, we used Franz diffusion cells as described above for the *in vitro* release studies. Skin obtained from female nude mice was clamped between the donor and the receptor chambers, with the respective formulations (paeonol cream 1.0 g, paeonol microsponge cream 1.0 g, or saturated aqueous solution 1 ml) placed gently in the donor chamber. The permeation membrane was dismantled after 4, 8, 12 and 24 h, respectively. The remaining cream outside the skin was carefully removed, and then the skin was weighed and cleaned with 10 ml distilled water each time (five times in total). The treated skin was then cut into small pieces, and the drug in the skin was extracted by homogenization with 5 ml methanol. Finally, the tissue samples were centrifuged at 10,000 rpm for 30 min. The supernatants were collected and analyzed using HPLC.

### *In vivo* microdialysis

Female Wistar rats weighing about 200 g were anesthetized with 10% chloral hydrate 0.35 ml/100 g, given as an intraperitoneal injection with supplementary injections of half the dose every 90 min if needed. For intra-dermal microdialysis, the abdominal fur of the rats was carefully shaved, and then the skin was incised over the dermis, followed by intra-dermal insertion of an introducer, assembled by inserting a stainless needle into the tubing. After placing the tubing into the dermis, the needle was withdrawn, followed by insertion of a microdialysis probe (CMA 20 Elite, 10 mm in membrane length, 20 kDa cut-off; CMA Microdialysis AB, Solna, Sweden) and then the tubing removed [24]. After placement, the probe was perfused with normal saline using a syringe pump (CMA 402; CMA Microdialysis AB, Solna, Sweden) at a constant flow rate of 5 µl/min. While the rat was still under anesthetized condition, the jugular vein was isolated. For plasma microdialysis, the microdialysis probe described above was placed in the jugular vein, and perfused with normal saline by the microdialysis pump at a constant flow rate of 5 µl/min. The system was equilibrated for 1 h, and then 1.0 g paeonol cream or paeonol microsponge cream was applied to an area of skin 3.14 cm^2^ in size. The intra-dermal and plasma dialysates were collected for HPLC analysis, using a refrigerated fraction collector (MAB 85; Microbiotech/se AB, Stockholm, Sweden) every 20 min for 12 h.

In order to obtain absolute tissue concentrations from dialysate concentrations, *in vivo* relative recovery (R*_in vivo_*) was carried out using retrodialysis prior to the microdialysis studies. In this experiment, one microdialysis probe was inserted into the dermis as indicated above, while the other probe was inserted into the jugular vein. Probes were perfused with 6.15 µg/ml paeonol at a constant flow rate of 5 µl/min, and the respective dialysates were collected at 20-min intervals and analyzed using HPLC. Recovery was determined from six consecutive dialysis samples per probe. R*_in vivo_* was calculated as:
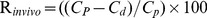
(4)where C_d_ is the drug concentration in the dialysate, and C_p_ is the initial perfusate concentration of paeonol [25]. Prior to the PK analysis, concentrations for all dialysate samples were corrected using the following equation:
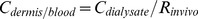
(5)where C_dermis/blood_ is the unbound drug concentration in the intradermal fluid or plasma.

### Statistical analysis

Data in all experiments are presented as means ± SD. Statistical differences were tested by one-way ANOVA and the independent samples *t*-test. *P*<0.05 was considered significant.

## Results

### Characterization of the microsponge formulation

**Figure 1:**
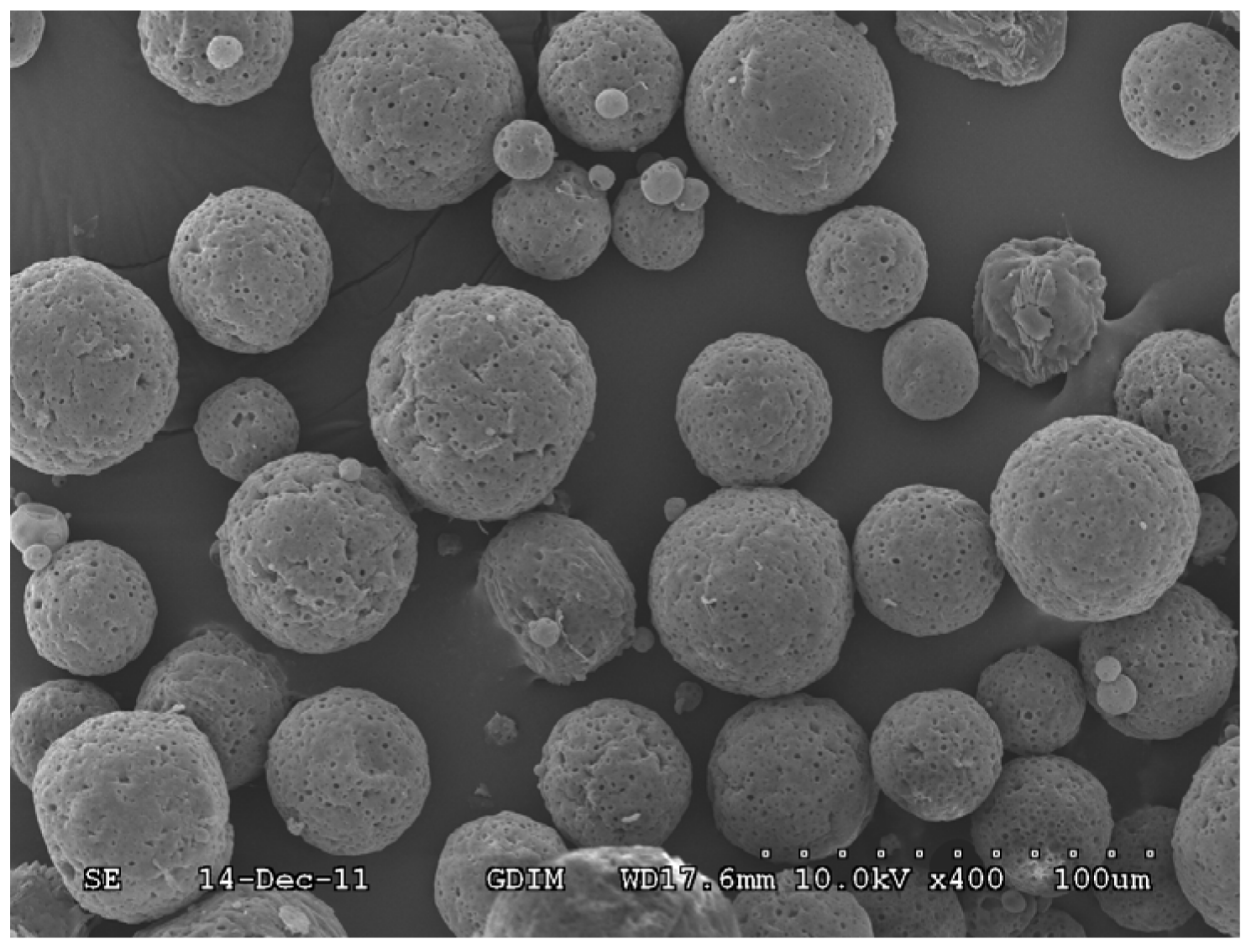
Scanning electron micrograph of the formed microsponges (original magnification ×400). *The length of all the dots is 100 µm*.

The morphology of the microsponges was studied by SEM. The typical shape and surface characteristics of the microsphere are shown in Figure 1. The microsponges were finely spherical and uniform in shape, and porous in nature, with no drug crystals on the surface.

The production yield, actual drug content, and loading efficiency was calculated according to equations 1 to 3. The production yield was 72.20±3.59% (n = 3), the actual drug content was 77.40±1.06% (n = 3) and the loading efficiency of the paeonol microsponge was 55.90±3.27% (n = 3).

Using the laser light-scattering technique, a particle size distribution map by volume of the paeonol microsponges was determined, which showed that the specific area, surface diameter and diameter by volume of the particles were 0.65 m^2^/g, 9.5 µm and 22.4 µm, respectively. The particle size distribution of d(0.1), d(0.5) and d(0.9) were 6.3 µm, 15.8 µm and 37.4 µm respectively, and the sorting coefficient was 0.569.

### *In vitro* release studies

**Figure 2:**
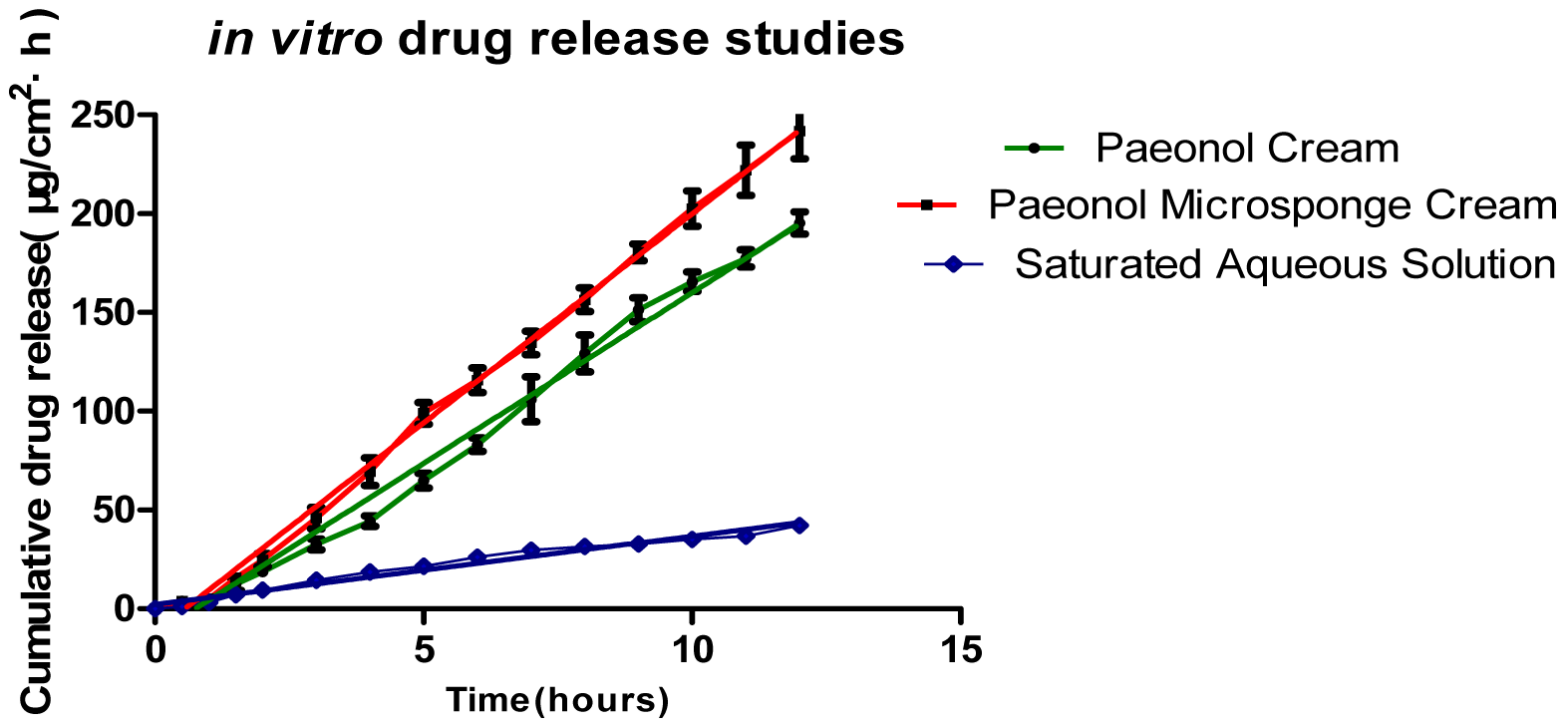
*In vitro* drug-release studies of 50 mg/g paeonol cream, 50 mg/g paeonol microsponge cream and 505.52 µg/ml saturated aqueous solution. *All data are shown as means ± SD (n = 3)*.

The *in vitro* release profiles of paeonol cream, paeonol microsponge cream, and saturated aqueous solution are shown in Figure 2. In order to have a better comparison between the release profiles of the three formulations, the slopes (flux) of the linear portion of the release profiles were calculated, as was the steady state flux (Jss), based on the cumulative amount of drug permeated per unit area plotted against time, and the estimated as steady state flux (J_ss_) (Table 1).

**Table 1:** Permeability characteristics of paeonol cream and paeonol microsponge cream in the *in vitro* drug-release studies.

Parameters	Paeonol microsponge cream	Paeonol cream	Saturated aqueous solution of paeonol
Flux (Jss), µg/cm^2^/h	21.17±0.284	17.29±0.312	3.46±0.091
Kinetics			
Zero order, R^2^	0.997	0.995	0.973
First order, R^2^	0.815	0.821	0.719
Higuchi, R^2^	0.912	0.902	0.968

*All data are shown as means ± SD (n = 3)*.

Based on these results, the *in vitro* release profile of paeonol microsponge cream could be best expressed by zero order kinetics, as the cumulative drug release *vs.* time were found to be linear (R^2^ = 0.997).

### *Ex vivo* drug-deposition studies

**Figure 3:**
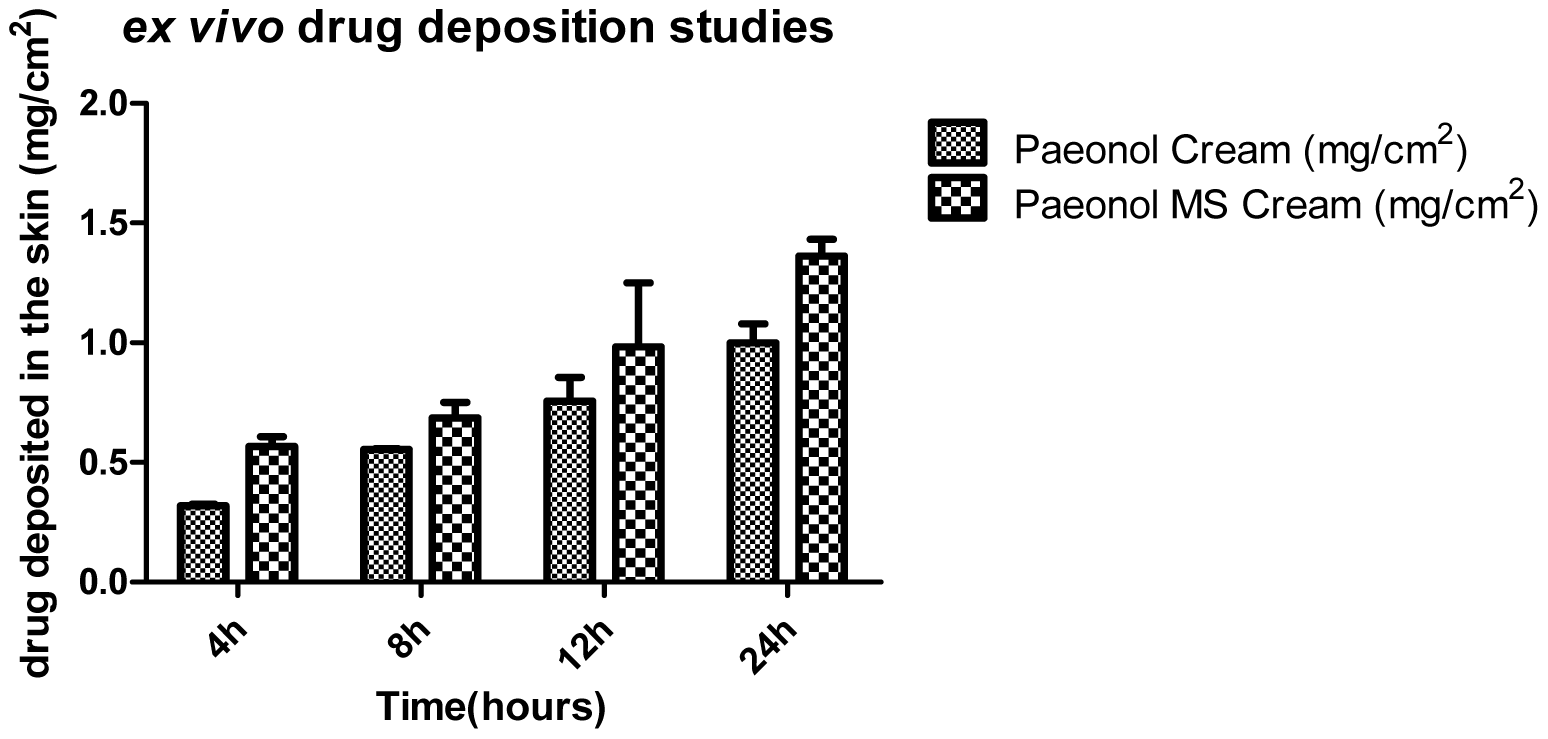
*Ex vivo* drug-deposition studies of paeonol microsponge cream (5%, 1 g) and paeonol cream (5%, 1 g). *All data are shown as means ± SD (n = 3)*.

The amount of paeonol deposited in the skin from different formulations at different time intervals was determined by HPLC (Table 2, Figure 3). As shown in Figure 3, the amount of paeonol deposited in the skin was much higher for the paeonol microsponge cream than for the paeonol cream, especially at 4 h (0.5675±0.0394 mg/cm^2^ *vs.* 0.3194±0.0091 mg/cm^2^) and 24 h (1.3627±0.0699 mg/cm^2^ *vs.* 0.9988±0.0801 mg/cm^2^). Therefore, the highest amount of paeonol deposited by the microsponge cream at the same dose was found after 24 h, indicating that the microsponge cream improved drug residence in the skin.

**Table 2:** The drug deposited in skin from paeonol microsponge cream and paeonol cream.

	Amount of drug deposited in skin (mg/cm^2^)	
Time(hours)	Paeonol microsponge cream	Paeonol cream
4	0.57±0.039	0.32±0.009
8	0.69±0.066	0.55±0.005
12	0.98±0.268	0.76±0.010
24	1.36± 0.070	1.00±0.080

*All data are shown as means ± SD (n = 3)*.

### *In vivo* microdialysis

The average percentage recovery of paeonol by the retrodialysis method over 120 min was 52.44±3.67% (n = 6) in the intradermal fluid and 51.70±3.75% (n = 6) in plasma. The dialysis membrane showed steady loss of paeonol for 120 min in the subcutaneous liquid and in plasma through the CMA-20 probe.

**Figure 4:**
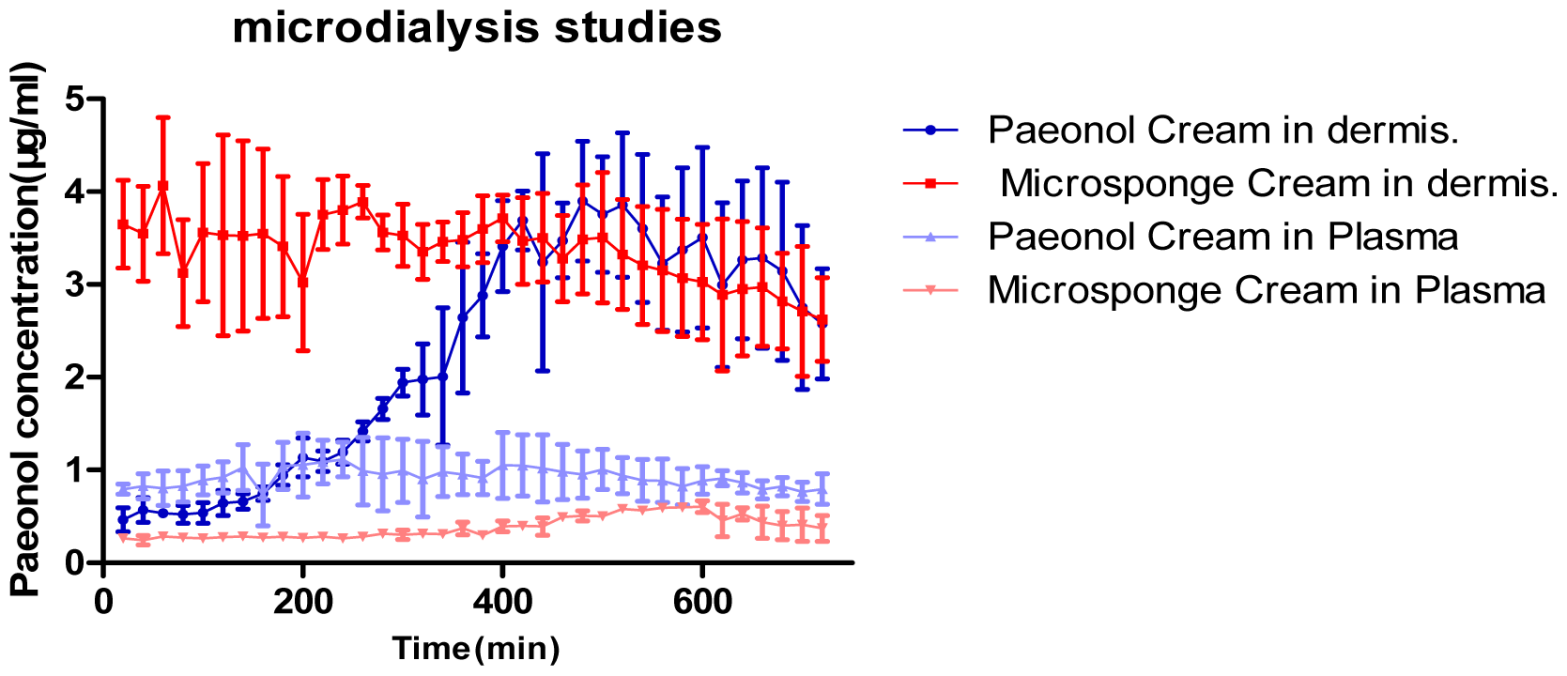
Paeonol concentration in intradermal liquid and plasma by *in vivo* microdialysis for paeonol cream and paeonol microsponge cream. *All data are shown as means ± SD (n = 3)*.

The corrected dialysate concentrations of paeonol in plasma and intradermal fluid after topical application of paeonol cream and paeonol microsponge cream over the time interval of 12 h are shown in Figure 4. The pharmacokinetic parameters for unbound paeonol were calculated (Table 3).

**Table 3:** Pharmacokinetic parameters of paeonol cream and paeonol microsponge cream by *in vivo* microdialysis.

	Intradermal fluid		Plasma	
Parameter	Paeonol cream	Paeonol microsponge cream	Paeonol cream	Paeonol microsponge cream
t_1/2_, min	548.6	935.1	449.7	214.0
T_max_, min	480	220	186.7	606.7
C_max_, µg/ml	4.060±0.5621	4.357±0.4765	1.145±0.2126	0.6359±0.0415
AUC_0∼t_, µg/ml/min	1587±308.0	2396±258.2	656.7±153.2	270.1±14.10
AUC_0∼∞_, µg/ml/min	3972±2875	6257±4636	1192±386.7	390.8±91.26

*All data are shown as means ± SD (n = 3)*.

As shown in Figure 4, the paeonol absorption was significantly higher with the paeonol microsponge cream than with the paeonol cream, which is in accordance with *in vitro* drug-release studies. Likewise, in the intradermal fluid, the area under the curve (AUC) for concentration versus time (AUC_0∼t_) value was much higher for paeonol microsponge cream (2396±258.2 µg/ml/min) than for paeonol cream (1587±308.0 µg/ml/min). Maximum time (T_max_) was 220 min for paeonol microsponge cream and 480 min for paeonol cream, while t_1/2_ for paeonol microsponge cream (935.1 min) was almost twice that of the paeonol cream (548.6 min). By contrast, the AUC_0∼t_ and C_max_ values of the paeonol microsponge cream were less than half that of the paeonol cream (270.1±14.10 µg/ml/min *vs.* 656.7±153.2 µg/ml/min and 1.145±0.2126/ml *vs.* 0.6359±0.04150 µg/ml, respectively).

## Discussion

### Characterization of microsponge formulations

SEM images showed that the paeonol microsponges were porous and had a spherical shape. The pores were caused by the diffusion of the solvent from the surface of the microsponges [22]. Thus, the volume of dichloromethane has a key role to play in the preparation of microsponges.

### *In vitro* release studies

It is considered that microsponge formulations are too large to pass through the stratum corneum, hence they would be expected to remain on the skin surface, gradually releasing their contents over time [22]. However, our results showed that preparing paeonol in microsponges could increase the permeation rate of the drug, compared with paeonol cream and saturated aqueous solution. This showed that the particle size has a significant effect on the drug release, and probably accounts for the discrepancy between our study and that of Jelvehgari et al [22], who used a pore size of about 300–400 µm, which is more than 10-fold larger than that used in our study. Our results indicate that microsponge formulations are able to increase the permeation rate of some liposolubility drugs formulated as small particles. Furthermore, the *in vitro* release profiles shown in Figure 2 indicated that paeonol microsponge cream was able to show a sustained release for up to 12 h compared with paeonol cream. The mechanism of drug release from microsponges may be associated with its porous surface, as this enables easy penetration of the release media and accessibility to the entrapped drug molecule [17]. As the release media penetrated into the porous of the microsponge, the drug was then dissolved into it, thus drug released from the microsponge. As the release media first gained access to the surface of the microsponge and then gradually into the internal, the drug release measured over the first few hours was due to the presence of non-encapsulated paeonol on the surface of the microsponge, then as the release media gained access to the pores, there was release of the drug entrapped in the pores [22], resulting in sustained drug release for up to 12 h.

### *Ex vivo* drug-deposition studies

Effective topical drug therapy requires a sufficient amount of drug to be taken up into the skin over a particular period of time to allow maximal pharmacological activity [2]. Thus, the larger amount of drug deposited in the skin from paeonol microsponge cream indicates greater drug bioavailability in the topical area, which is in agreement with an earlier report [2]. The higher drug retention by the paeonol microsponges for all time points may be partly explained by the occlusive effect, as the microparticles produced a film on the skin surface, which reduced transepidermal water loss. And then increased the hydration state of the stratum corneum, and finally leading to increased drug penetration into the skin [26], [27]. Moreover, the high lipophilicity of the drug microsponge formulation prevented drug diffusion from the skin into the receiver fluid [28], thus maintaining efficacious local drug levels for a long period of time.

### *In vivo* microdialysis

As shown in Table 3, the observed T_max_ for paeonol microsponge cream was 220 min, compared with 480 min for paeonol cream. This may be due to the microsponge formulation producing an occlusive layer on the skin surface and thus reduced the transepidermal water loss and leading to high drug penetration [29], which was in accordance with the *in vitro* release studies. The average t_1/2_ for the paeonol microsponge cream and paeonol cream was 935.1 min and 548.6 min, respectively, thus the microsponge formulation showed good distribution into the dermis. As shown in Figure 4, the drug concentration of paeonol microsponge cream was very stable compared with that of paeonol cream, showing that the microsponge could act as a drug reservoir within the upper layers of the stratum corneum, allowing the drug to be released in a controlled fashion [1]. For treating skin disease, the drug (in this case, paeonol) should stay in the treated areas and there should be no absorption to other areas. The AUC measured by microdialysis represents the total amount of drug that penetrated through the stratum corneum, into the epidermis and finally into the dermis after 12 h. Therefore the higher values of AUC_0∼12 h_ for paeonol microsponge cream in the subcutaneous liquid showed that the microsponge formulations could deliver significant amounts of paeonol to the dermis, thus leading to high drug bioavailability, but with high inter-individual variability. However, the lower values of AUC_0∼12 h_ and C_max_ for paeonol microsponge cream in plasma showed that the amount of drug absorbed into the plasma was not significant, thus this should result in reduced side-effects. This reduced absorption may be due to the high lipophilicity of the drug formulation preventing drug diffusion from the skin into the interstitial fluid [28]. The results indicated that preparing paeonol as a microsponge formulation may be an effective drug-delivery system for skin targeting in treating skin disease.

Major limitations of the microdialysis method are the low recoveries of molecules with large molecular weights (>20 kDa) and high lipophilicity or high protein binding of some drugs [30]. To determine the appropriate perfusate for paeonol, we also measured the average recovery and protein binding. The hydrophilicity (2.21 of partition coefficient), low molecular weight (166.18), high recovery (52.44% in intradermal fluid and 51.70% in plasma) and low protein binding (33.01±5.75) of the drug, showed microdialysis method was particularly appropriate for paeonol with saline as perfusate.

**Figure 5:**
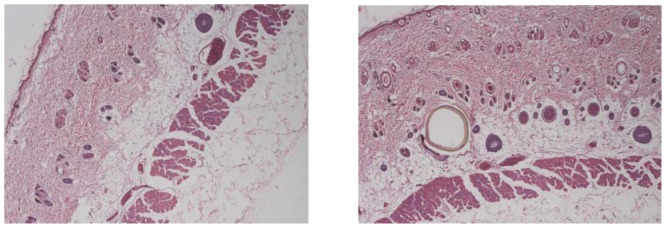
Histological analysis of the probe depth in the dermis (×40). A. dermis without probe inserted into it;B. dermis with a probe inserted into it.

There are controversial results regarding the influence of the actual position of the probe on recovery. The different probe depth into the dermis may influence the drug concentration in intradermal fluid. All the probes were inserted into nearly the same depth by the same person and the histological analysis was used to confirm that (Figure 5). And Esther et al. has reported that nearly consistent depths can be achieved if the probes are inserted by the same trained person [31]. However the exact value in the dermis depth was not available. But in a skin penetration study of salicylic compounds, there was no significant correlation between probe depth and drug concentration in the range from 0.7 to 1.1 mm [25]. The same conclusion had been found in the report of Muller et al. and Hegemann et al. [31]. And Esther had also reported that there would be no significant correlation between probe depth and drug concentration when the variation in probe depth was very small [31].

## Conclusions

In this study, we first found that the microsponge delivery system could not only increase paeonol permeation rate but also minimize transdermal penetration of the drug into the body, which should increase drug bioavailability at the level of the skin and reduce side-effects when treating skin disease. Additionally, microsponges were able to improve the drug residence in skin and allowed sustained drug release for up to 12 h, resulting in a long active time for the drug in topical treatment of the skin. These properties indicate that a microsponge delivery system could be a useful strategy for a new generation of pharmaceutical and cosmetic treatments. However, the level of drug distribution in the stratum corneum, epidermis, and dermis and the mechanism of drug penetration is still unknown, thus further studies into these aspects are needed.
